# Innovative concepts in diet therapies in disorders of gut–brain interaction

**DOI:** 10.1002/jgh3.70001

**Published:** 2024-07-18

**Authors:** Daniel So, Caroline Tuck

**Affiliations:** ^1^ Department of Gastroenterology Monash University and Alfred Health Melbourne Australia; ^2^ Department of Nursing and Allied Health Swinburne University Hawthorn Australia

**Keywords:** DGBI, dietary fiber, enzymes, IBS

## Abstract

Diet therapy in disorders of gut–brain interaction (DGBI) is rapidly advancing, with accumulating evidence to support two innovative therapies—manipulation of dietary fibers and enzyme supplementation—that target specific DGBI pathophysiology and modulate digestion. Dietary fibers escape digestion in the upper gastrointestinal tract and can influence gut function by impacting digestion, improving laxation, and interacting with the microbiota. A more nuanced understanding of different fiber types and their ability to impact gut function in highly specific ways has shown that fibers can impact distinct gut symptoms and pathophysiology. By considering their functional characteristics of bulking, gel‐forming, and fermentability, restriction or supplementation of specific fibers can offer clinical value in DGBI. Similarly to fiber specificity, emerging evidence suggests that supplemental digestive enzymes may be targeted to known food triggers with consideration that enzymes are substrate specific. Limited evidence supports use of lactase to target lactose, and α‐galactosidase to target galacto‐oligosaccharides. Application of enzymes during manufacturing of food products may prove to be an additional strategy, although evidence is scant. Both innovative therapies may be utilized in isolation or in combination with other diet and nondiet therapies. Implementation can be guided by the principles that fiber modulation can be targeted to specific symptomology or requirement for alterations to gut function, and digestive enzymes can be targeted to known food triggers. This review aims to summarize recent literature of these two innovative concepts and provide practical suggestions for their implementation in clinical practice.

## Introduction

Disorders of gut–brain interaction (DGBIs) are heterogeneous group of over 30 gastrointestinal disorders characterized by chronic or recurrent gastrointestinal symptoms occurring in the absence of organic disease.^1^ These disorders do not contribute directly to mortality, but are associated with impaired quality of life^2^ and psychological comorbidity.^3^ The characterizing symptoms of DGBIs include any combination of disruptions to brain–gut communication, dysfunctions to the gut microbiota, disordered motility, visceral hypersensitivity, and altered mucosal and immune function.^1^ Approximately 40% of individuals worldwide are affected by DGBIs, with irritable bowel syndrome (IBS), functional constipation, and functional dyspepsia (FD)—the most extensively researched disorders.^4^

Diet therapies are being increasingly utilized in the management of symptoms in DGBIs, both as standalone therapy and as part of integrated care.^5^ For example, a low FODMAP (fermentable oligo‐ di‐mono‐saccharides and polyols) diet is now applied as a frontline therapy in IBS,^6^ with a substantial evidence base demonstrating efficacy and scientific premise.^7^, ^8^ While mechanistic evidence for the low FODMAP diet initially focused on acute reductions in colonic fermentation, more recent studies have shown effects related to gut–brain dysregulation,^9^ modulation of pain signaling,^10^ and altered permeability.^11^, ^12^, ^13^, ^14^ Additionally, mechanistic evidence of the role of diet beyond FODMAPs suggests that local immune response to dietary antigens triggered by bacterial infection may lead to food‐induced symptoms^15^ requiring further exploration, as reviewed recently.^16^ Beyond these, evidence is accumulating for two innovative supplemental diet therapies in DGBI: targeting specific pathophysiology through manipulation of dietary fibers and modulating digestion through the use of digestive enzymes. These innovative therapies may be used in conjunction with or instead of other diet‐ and nondiet‐focused therapies.

## Precision medicine: Manipulation of dietary fibers

Dietary fibers describe a group of carbohydrates that are intrinsically found in plant‐based foods. Fibers escape digestion in the upper gut and can affect gut function in a variety of ways, from slowing the rate of gastric emptying to improving laxation.^17^ Supplementation with dietary fibers is commonly used as a diet therapy to improve overall gut function in otherwise healthy individuals with inadequate fiber intake,^18^ with different types of fiber used interchangeably. However, in individuals with DGBIs, fiber supplementation is less widely applied, with evidence from clinical trials showing its application led to modest or no effects.^19^

This lack of benefit can be attributed to how fibers have traditionally been applied in this patient group, which is primarily as a laxation agent to improve symptoms by normalizing disordered motility, which does not address the other pathophysiology of DGBIs that were not recognized at the time.^20^ Indeed, the number of potential therapeutic targets in DGBI has expanded as it is now recognized that their pathophysiology extends beyond disordered motility. Coincident with these advancements is a movement toward more nuanced understanding of fibers. Specifically, an appreciation that fibers are not interchangeable, and that different types of fiber capable of impacting gut function in highly specific ways. Together, this has led to renewed interest in how fibers can be manipulated, through supplementation and restriction, for therapeutic benefit in DGBIs.

### Fiber types

The predominant advance in our understanding of fiber is that the concepts of “soluble” and “insoluble” fibers have little bearing on their physiological effects. Whether a fiber solubilizes in water offers little insight to their behavior in the gut. For example, both fructo‐oligosaccharide (FOS) and hydrolyzed acacia gum are soluble but have different effects in the colon: FOS is readily fermented while the acacia resists fermentation,^17^ and thus, these “soluble” fibers are not interchangeable. Instead, there is increasing recognition that fibers should be described or categorized according to their functional characteristics: capacity to undergo fermentation by the microbiota, to form viscous gel structures, and ability to bulk the stool. The shift toward a functional paradigm has been discussed in recent reviews^17^, ^18^, ^21^ but not yet adopted in guidelines,^22^, ^23^, ^24^ although functional effects beyond solubility are beginning to be recognized.^22^

#### Fermentable fibers

Some types of fiber are fermented by the resident bacteria upon reaching the colon, which generates gases (e.g., hydrogen and methane) and short‐chain fatty acids while modulating microbiota composition. Fibers that can resist fermentation retain their other characteristics and have little direct effect on the microbiota. The rate of fermentation is a particularly important consideration for fermentable fibers, as the speed of their breakdown impacts the rate at which the metabolites generated are released to the lumen, with the rapid release of gases capable of stretching mechanoreceptors that line the lumen. Rapidly‐fermented fibers include fructans and galacto‐oligosaccharides (GOS); moderate to slowly‐fermented fibers include beta‐glucans and resistant starch 2.^17^

#### Gel‐forming/viscous fibers

Gel‐forming or viscous fibers predominantly act in the upper gut. These fibers form a gel structure to slow the rate of gastric emptying, and mediates postprandial rises in blood glucose by decreasing the rate of nutrient absorption in the small intestine. In the colon, the gel structures formed can either contribute to stool bulk or are degraded by the microbiota, depending on capacity to resist fermentation. Viscous fibers that are fermentable include beta‐glucans; viscous fibers that are poorly fermented include psyllium.^17^

#### Bulking fibers

Bulking fibers predominantly act in the colon. These fibers contribute to stool bulk drawing and retaining water in their matrices, and by stimulating fluid secretion into the lumen through particulate effects. These fibers are still referred to as “insoluble” or “particulate” fibers.^18^, ^21^, ^25^ Bulking fibers are generally poorly fermented and rich in cellulose; wheat bran is fiber composite with strong bulking effects but is also partially fermentable.^17^

### Applications of fiber manipulation in DGBIs

The broadening of therapeutic targets in DGBIs has provided more opportunities for manipulation of specific fibers to be utilized as a diet therapy (summarized in Table 1). Examples of the ways that fibers can influence specific symptoms include normalizing stool form and laxation, limiting colonic fermentation to minimize distension of the lumen, and manipulating colonic fermentation to modulate the gut microbiota.

**Table 1:** Summary of fiber types, applications, and evidence in DGBI (evidence specifically refers to IBS unless otherwise indicated)

Fiber type	Examples	Application	Potential benefit to DGBI	Evidence
Rapidly fermented	Fructans, GOS	Restriction (low FODMAP diet)	Attenuate luminal distension	Reduces gut symptoms in IBS and FD. Modulates microbiota composition (Bifidobacteria abundance reduced)
		Supplementation; monotherapy	Modulate microbiota (enhancing overall fermentation)	No benefit to gut symptoms, flatulence increased with fructans. Modulates microbiota composition (Bifidobacteria & Lactobacilli abundance increased)
		Supplementation; adjunct to low FODMAP diet	Modulate microbiota (enhancing overall fermentation) while attenuating luminal distension	No impact on gut symptoms. No impact on microbiota composition
Slowly fermented	Resistant starch 2, beta‐glucan	Supplementation	Modulate microbiota (enhancing overall fermentation)	Limited data
Poorly fermented, bulking, and viscous	Psyllium, nopal	Supplementation; monotherapy	Improve laxation	IBS: inconsistent effects on laxation and gut symptoms; Improvements on laxation not shown to occur in parallel to impacts on symptoms Constipation: efficacious effects on laxation and overall symptoms
Poorly fermented, bulking, non‐viscous	Sugarcane bagasse	Supplementation; adjunct to low FODMAP diet	Improve laxation	No impact on gut symptoms Improves laxation in patients with constipation‐predominant symptoms as adjunct therapy
Partially fermented, bulking	Wheat bran	Supplementation	Improve laxation	Inconsistent effects on laxation and gut symptoms. Poor tolerability reported
Combination: rapidly fermented with poorly fermented, bulking and viscous	Inulin with psyllium	Supplementation; monotherapy	Modulate microbiota (modulating regional fermentation) and improve laxation	Impact on symptoms unclear. Fermentation of inulin in proximal attenuated
Combination: slowly fermented with poorly fermented, bulking and nonviscous	Resistant starch 2 with sugarcane bagasse	Supplementation; adjunct to low FODMAP diet	Modulate microbiota (modulating regional fermentation) and improve laxation, while attenuating luminal distension	No impact on gut symptoms. Site of fermentation shifted toward distal colon

There is substantial evidence demonstrating that limiting colonic fermentation leads to a therapeutic effect in IBS, with emerging evidence suggesting it may also have utility in FD.^26^, ^27^ This has been consistently demonstrated in trials of the low FODMAP diet,^7^ where the intake of rapidly‐fermented oligosaccharide fibers (fructans and GOS), together with incompletely‐absorbed short‐chain carbohydrates, are restricted.^8^ As a result, the fermentation of contents entering the colon and subsequent release of metabolites, namely gases, into the lumen, are limited, reducing the gut symptoms generated through distension of the lumen in the setting of visceral hypersensitivity.^8^ Limiting colonic fermentation through this approach has minor effect on microbiota composite,^28^ which has been shown to be restored with gradual re‐introduction of fermentation.^29^

Several different fiber types have been utilized to target laxation and/or improve symptoms in IBS: psyllium, wheat bran, nopal fiber, and sugarcane bagasse. Psyllium, nopal, and sugarcane bagasse are poorly fermented, while wheat bran contains a fraction of fructan^30^ and is thus partially fermented.^31^ Supplementation with psyllium and wheat bran (6–30 g/day) led to inconsistent effects on indices of laxation (stool form, stool frequency, transit time) as well as gut symptoms, although notably, beneficial changes in laxation did not occur in parallel with improvements in symptoms.^17^ However, supplementation with psyllium was tolerated more consistently than wheat bran,^19^, ^32^ presumably due to the fermentability of the latter. Supplementation with nopal fiber (10–30 g/day) with typical dietary intake led to a greater rate of overall symptom relief compared with placebo but had little effects on the indices of laxation.^33^ Sugarcane bagasse, supplemented on top of a low FODMAP diet at 10 g/day, led to specific changes in laxation but did not improve or worsen symptoms compared with the base diet therapy. However, the supplemental fiber produced specific benefits for patients with constipation‐oriented symptoms: hastening colonic transit rate and normalizing stool form.^34^ Taken together, these data suggest that supplementation with poorly‐fermented fiber alone is well‐tolerated but has varied effects on symptoms and laxation, but may have specific utility when used in conjunction with other therapies. In constipation, the differential effects of specific fibers have been more clearly demonstrated, with meta‐analysis showing that supplementation with psyllium led to improvements in both symptoms and indices of laxation, whereas fermentable fibers (fructans, GOS, and polydextrose) had little effects.^35^

Supplementation with fermentable fibers in DGBIs have been utilized to modulate the gut microbiota in different ways. These include supplementation with habitual diet to promote the growth of purportedly beneficial bacteria as a means of modulating symptoms in IBS and FD and concomitant supplementation with a low FODMAP diet to offset the microbiota effects of the diet in IBS. In IBS and FD, supplementation with fermentable fibers (3–24 g/day) led to increased Bifidobacteria abundance.^36^ However, these effects did not occur in parallel with changes in overall symptoms or abdominal pain, with fructan supplementation leading to greater flatulence.^36^ Concomitant supplementation of fermentable fiber (1.4 g/day beta‐GOS) with a low FODMAP diet did not impact response to the base diet therapy, but had no impact on the microbiota either.^37^ This suggests that supplementation of fermentable fibers alone offers minimal symptomatic benefit in DGBIs and that low doses do not impact the microbiota in IBS.

The use of fiber combinations in IBS is an emerging concept explored using psyllium with inulin (20 g each) in an acute challenge, and sugarcane bagasse (10 g/day) with resistant starch 2 (12 g/day) with a low FODMAP diet intervention. Both combinations led to a change in fermentation pattern: the addition of psyllium to inulin attenuated the rise in colonic gas produced compared with inulin alone, as measured via magnetic resonance imaging^25^; and the combination of sugarcane bagasse to resistant starch 2 pushed fermentation toward the distal colon, as measured using telemetric capsules^38^. These effects, which can now be assessed and quantified through the emergence of these technologies and techniques, can be used to guide more precise applications of fibers to modulate the microbiota in DGBIs and beyond.

## Modulating digestion: Digestive enzymes

As compared with fiber modification, which can be targeted toward specific symptoms, digestive enzymes may be targeted toward specific known dietary triggers. Dietary triggers are commonly reported by patients with a range of DGBIs including FODMAPs, dairy, gluten, and wheat.^39^, ^40^ While dietary modifications such as the FODMAP diet are efficacious, they are not without side effects such as proposed negative effects on nutritional adequacy, microbiota composition, and risk of disordered eating.^41^ To mitigate these effects, oral preparations or food application of digestive enzymes offer a potential strategy to improve food tolerance and liberalize restrictive diets. Given the specificity of enzymes to substrate, a range of enzymes have been investigated for their effect on known food triggers including various FODMAP subgroups and gluten.

### Oral enzymes

#### Lactase

In the absence of sufficient endogenous lactase, the disaccharide lactose can be hydrolyzed by commercially available lactase enzyme supplements. The enzyme is taken with the first mouthful of lactose containing foods and additional enzyme may be taken at the end of the meal,^42^ although the timing of enzyme consumption has been poorly studied. Oral lactase has been shown to decrease both breath hydrogen and symptom response,^43^, ^44^, ^45^ with an 88% reduction in breath hydrogen (*p* < 0.01), and a dose‐dependent improvement in symptoms (*p* < 0.0001 vs. placebo) when given with 25 g lactose challenge.^46^ However, higher doses of lactose, such as 50 g, appear to overwhelm the ability of the enzyme.^43^ Despite heterogeneity in timing, lactose and enzyme dosing across studies making comparisons difficult, the enzyme is generally recognized as a valid therapy for targeting lactose‐related gastrointestinal symptoms.^47^

#### Xylose isomerase

Xylose isomerase catalyzes the reversible isomerization of glucose and fructose, and is used in the food industry for the production of high fructose corn syrup.^48^ Breath hydrogen and abdominal pain were reduced in a single study comparing xylose isomerase to placebo with 25 g fructose test solution in 65 patients with abdominal symptoms and fructose malabsorption.^49^ However, low symptom scores were reported throughout the study; hence, it is unknown how effective the enzyme is in those with more severe symptoms. It is also unknown how the enzyme response may differ when applied to whole foods. Additionally, given the osmotic action of slowly absorbed fructose,^50^ the rate at which xylose isomerase can convert the fructose to glucose within the small intestinal may be key. If full conversion occurs in the proximal small intestine, then improved symptom control may be achieved, but if conversion of fructose takes place along the length of the small intestine, the osmotic action of the yet‐to‐be‐converted fructose may still be enough to create luminal distention, resulting in symptoms.

#### Alpha‐galactosidase

Another commercially available FODMAP‐specific oral enzyme is α‐galactosidase, an enzyme targeting GOS present in legumes, nuts, and soy products. Reduced symptoms in patients with IBS deemed as GOS‐sensitive (those who had symptoms with placebo) occurred in a cross‐over study where the enzyme was given with 3 days of high GOS foods.^51^ An additional study in children with gas‐related symptoms also suggested benefit of the enzyme.^52^ In contrast, two studies in IBS patients showed no effect of enzyme when given with habitual diet^53^ or with high FODMAP meals containing excess fructose, polyols, fructan, and GOS.^54^ The lack of symptom improvement in these studies may be attributed to the presence of other FODMAPs within the diet, especially given the enzyme has been shown *in vitro* to have specificity to GOS.^55^ The differing effects of the enzyme across these dietary designs highlight that enzyme therapy must be targeted appropriately to foods containing the substrate of interest, which in this case would be targeting high GOS foods in patients with known GOS‐sensitivity.

#### Fructanase and combined FODMAP enzymes

Given the frequent co‐existence of GOS and fructans within commonly consumed foods such as wheat and rye, an oral fructanase (or inulinase) that could be consumed combined with α‐galactosidase is theoretically an attractive therapeutic option. *In vitro* studies using simulated gastrointestinal digestion show the release of fructose when inulinase is used with a test meal containing foods high in fructan.^56^ However, given excess fructose is a FODMAP itself, it is unclear how this may impact on symptom induction when applied in patients with IBS. Further *in vitro* studies have also investigated combination preparations containing enzymes to degrade lactose, fructan, and GOS, which have suggested the majority of excess fructose is reabsorbed in the simulated small intestine.^57^, ^58^ When applied to the simulated colon with healthy or IBS‐D donor feces, reductions in gas and short‐chain fatty acids occur.^57^, ^58^ How these *in vitro* results will apply in patients experiencing symptoms with high oligo‐saccharide foods is not known. Despite this, many enzyme‐mix preparations are now readily available on the market, hence until further clinical data are obtained, they should be used on a cautious trial‐and‐error basis and side effects carefully monitored.

#### Sucrase

Sucrose has not traditionally been considered as a FODMAP given it is usually rapidly hydrolyzed and absorbed in the human small intestine. However, given decreased symptom relief in patients with IBS carrying the sucrase‐isomaltase (SI) hypomorphic variants,^59^ it has been suggested that sucrose may act as a FODMAP in a subgroup of patients with IBS. Unfortunately, limited data exist for the use of sucrase in IBS. One study of 28 children with congenital sucrase‐isomaltase deficiency given 2 g/kg of sucrose showed reduced breath hydrogen and symptoms.^60^ Data in adults are currently limited to case reports suggestive of enzyme efficacy in IBS,^61^ with benefits proposed. Hence, more data are needed to understand the efficacy of sucrase in IBS.

#### Glutenase

Oral glutenases have been developed from plant and microbial peptidases, with multiple enzyme preparations available on the market. However, their target group is unclear, with some suggested for accidental or intentional gluten intake in coeliac disease, compared with others for IBS or non‐coeliac gluten/wheat sensitivity.^62^ A small number of studies performed in patients with coeliac disease, self‐reported non‐coeliac gluten sensitivity, and healthy controls have been performed, although study design has been heterogeneous in terms of duration and dose of enzyme and gluten used. In healthy controls, AN‐PEP enzyme was able to degrade a majority of 4 g gluten challenge in the stomach, suggesting its effect would be mostly complete prior to entry into the small intestine.^63^ In 34 patients with coeliac disease, the enzyme attenuated small intestinal injury from 2 g/day gluten for 6 weeks and provided a trend toward reduced symptoms, although 3 of 16 receiving enzyme had reductions in mean villus height to crypt depth ratio.^64^ Given the limited literature, differences in the dose of enzyme and gluten used, as well as unclear target group (i.e., coeliac disease vs. IBS), more data are required to understand the clinical application of glutenases.

### Oral enzyme optimization

Strategies to optimize enzyme therapies in IBS can be extrapolated from pancreatic enzyme replacement studies. First, pancreatic enzymes appear to be susceptible to deactivation through the stomach. Hence, formulations of pancreatic enzyme therapy either require larger dosages to counteract the loss, assuming a percentage of the dose can survive the stomach, or the use of enteric‐coatings to protect the enzymes during transit through the stomach.^65^ Second, as the aim is to optimize intra‐gastric mixture of the enzyme with the meal to allow simultaneous emptying from the stomach into the small intestine, the timing of enzyme consumption has been investigated.^66^ Although the exact recommendations vary, benefits of splitting the enzyme dosage between the beginning of the meal, mid‐meal, and immediately following the meal have been reported.^67^, ^68^, ^69^ While these strategies have not been specifically studied for targeting oral enzyme use in IBS, given the similar concept they likely have relevance in IBS.

### Enzyme addition to food products

An alternative to consuming enzymes at time of food consumption is to add the enzyme during food preparation or manufacturing. Similar to that seen where sourdough culture can reduce the FODMAP content of breads,^70^ addition of enzymes can reduce the FODMAP composition of the end product. Dairy products containing lactase are already saturated within the marketplace in some countries, including Australia. However, a systematic review suggested only 4 of 11 studies reported improved symptoms with lactose‐free compared with lactose containing dairy products.^71^ Recent innovations in food industry have shown >90% fructan reductions in rye sourdough with inulinases,^72^ and >90% GOS reductions in pea protein with α‐galactosidase.^73^ The benefits of enzyme‐aided FODMAP reduction in food products is that the enzyme action is specific, as compared with other techniques such as fermentation that may alter the food characteristics more broadly.^74^ Future considerations to optimize enzyme use in food industry will be optimizing pH and temperatures, enzyme dosing, and combination techniques such as combining with fermentation as a strategy to reduce excess fructose produced by enzymes such as inulinases.^74^ Clearly, further human data in patients experiencing symptoms are needed to understand their clinical relevance. But given the uptake of the FODMAP diet for IBS symptom management, it is likely enzyme‐modified food products will become increasingly available.

## Clinical application

**Figure 1:**
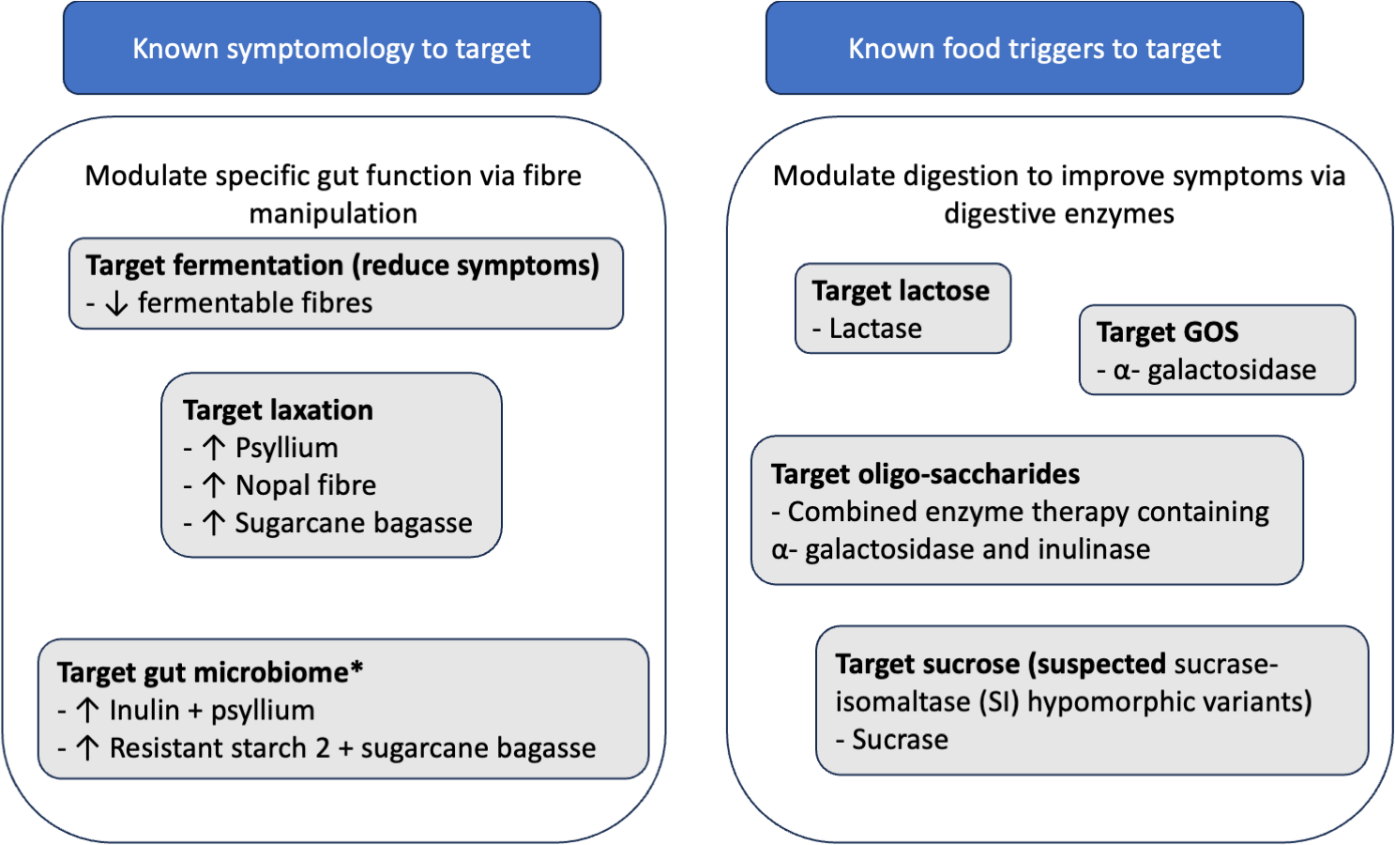
Summary of proposed application of innovative concepts in disorders of gut–brain interaction. *Evidence supporting clinical benefit limited.

Both dietary fibers and digestive enzymes offer potential therapeutic strategies that may be utilized in combination with other dietary therapies such as a FODMAP diet, or in some cases as standalone therapies. The challenge for the clinician is to know how to prioritize which therapy to target. Table 2 provides some suggested ideal candidates for the various fiber modification and digestive enzyme strategies. Given that fiber modulation can be targeted to specific symptomology or requirement for alterations to gut function, and digestive enzymes can be targeted to known food triggers, their implementation may be guided by these principles, that is, in a patient with known food triggers such as milk containing lactose or legumes containing GOS, enzyme therapy using lactase or α‐galactosidase respectively may be best placed as a therapeutic strategy. As compared with a patient with constipation‐predominant symptoms commencing a low FODMAP diet, adjunctive sugarcane bagasse supplementation may be a more appropriate therapeutic option. While both innovative therapies need not be used in isolation, their order of implementation may be guided in this way, as shown in Figure 1. While some emerging literature exists for these innovative concepts, more data are needed to further support their use and strengthen evidence base for more routine clinical application. As such, if used in clinical practice, careful monitoring for symptom response and side effects is needed. Given the range of diet‐induced mechanisms for symptom induction in DGBI,^16^ other food triggers may need to be considered when fiber and enzyme therapies are unsuccessful at improving symptom response.

**Table 2:** Summary of evidence and clinical application for innovative oral supplemental therapies in IBS

	Common forms available	Proposed mechanism of action	Evidence	Ideal candidates	Guideline for implementation
Fiber therapy	Psyllium, nopal	Improve laxation without luminal distension	Supplementation with habitual diet (6–30 g) well‐tolerated but effects on symptoms and laxation inconsistent	Patients with disordered motility	Implement as monotherapy; cautious trial‐and‐error approach warranted, with monitoring for symptom response, as data inconsistent
	Sugarcane bagasse	Improve laxation without luminal distension	Supplementation at 10 g/d did not impact symptoms but normalized laxation in patients with constipation‐predominant symptoms	Symptomatic patients with constipation‐predominant symptoms	Cautious implementation in conjunction with low FODMAP diet, monitoring for symptom response.
Enzyme therapy	Lactase (oral preparation)	Cleaves the disaccharide lactose into monosaccharides (glucose and galactose)	Reduced symptoms and breath hydrogen at doses up to 25 g shown. No benefit at larger doses (50 g).	Known lactose intolerance	Implement after FODMAP re‐challenges are conducted and individual lactose‐sensitivity is known, monitor symptom response.
	Xylose Isomerase	Catalyzes the reversible isomerization of glucose and fructose	Limited data suggesting benefit, but more data needed in symptomatic populations.	Insufficient data available.	Limited data available, hence cautious trial‐and‐error approach could be considered with monitoring for side effects.
	Alpha‐galactosidase (oral preparation)	Hydrolyzes α (1–6) linkages to release galactose	Shown to be effective in one study in IBS, only when targeted to high GOS foods. No benefit when used with usual diet or foods containing other FODMAPs, for example, fructan.	Target to those who are known to be GOS‐sensitive. May be beneficial in vegetarian/vegan patients using a FODMAP diet. Pediatric populations may benefit.	Consider trial, after FODMAP re‐challenges are conducted and individual GOS‐sensitivity is known, monitor for symptoms response.
	Fructanase	Hydrolyzes 2,1‐linked b‐glycosidic Fru–Fru bonds and terminal Glu–Fru bond.	*In vitro* data suggests ability to convert fructan to excess fructose, with suggestion that excess fructose is reabsorbed. However, human data in symptomatic population needed.	Insufficient data available.	Limited data available, hence cautious trial‐and‐error approach could be considered with monitoring for side effects.
	Combined FODMAP enzymes	Targets multiple FODMAP subgroups as per individual enzymes above	*In vitro* data suggests ability to hydrolyze various FODMAP subgroups, but human data in symptomatic population needed.	Insufficient data available.	Limited data available, hence cautious trial‐and‐error approach could be considered with monitoring for side effects.
	Sucrase	Sucrose may act as a FODMAP in a subgroup of patients with sucrase‐isomaltase (SI) hypomorphic variants	Limited data in children and case reports in adults suggesting benefit. However, further data in IBS populations needed.	Insufficient data available, may be relevant for those with sucrase‐isomaltase (SI) hypomorphic variants.	Limited data available, hence cautious trial‐and‐error approach could be considered with monitoring for side effects.
	Glutenase	Degrades whole gluten and gluten peptides into non‐immunogenic residues	Limited data in small numbers of coeliac disease, non‐coeliac gluten/wheat sensitivity and healthy controls.	Insufficient data available, with unclear target group.	Insufficient data available, with unclear target group.

## Conclusion

As we gain a better understanding of the dietary fiber and how different fibers impact gut function, it is clear that manipulating their intake can offer clinical value in the management of DGBIs, with different fibers and fiber types capable of offering highly specific utility to specific patients and endpoints. Similarly, further data on the efficacy of digestive enzymes as oral supplementation, or added to food, will enable clinicians to better target their use to specific patient cohorts. Further advancements in the understanding DGBIs may provide more opportunities for these innovative therapies, while development of novel techniques and devices will be critical for measuring the effects and understanding their mechanisms.

## References

[1] Drossman DA , Tack J . Rome foundation clinical diagnostic criteria for disorders of gut‐brain interaction. Gastroenterology. 2022; 162: 675–679. 34808139 10.1053/j.gastro.2021.11.019

[2] Knowles SR , Skvarc D , Ford AC *et al*. Negative impact of disorders of gut‐brain interaction on health‐related quality of life: results from the Rome foundation global epidemiology survey. Gastroenterology. 2023; 164: 655–668.e610. 36565940 10.1053/j.gastro.2022.12.009

[3] Staudacher HM , Black CJ , Teasdale SB , Mikocka‐Walus A , Keefer L . Irritable bowel syndrome and mental health comorbidity – approach to multidisciplinary management. Nat. Rev. Gastroenterol. Hepatol. 2023; 20: 582–596. 37268741 10.1038/s41575-023-00794-z PMC10237074

[4] Sperber AD , Bangdiwala SI , Drossman DA *et al*. Worldwide prevalence and burden of functional gastrointestinal disorders, results of Rome foundation global sudy. Gastroenterology. 2021; 160: 99–114.e113. 32294476 10.1053/j.gastro.2020.04.014

[5] Chey WD , Keefer L , Whelan K , Gibson PR . Behavioral and diet therapies in integrated care for patients with irritable bowel syndrome. Gastroenterology. 2021; 160: 47–62. 33091411 10.1053/j.gastro.2020.06.099

[6] Gibson PR . The evidence base for efficacy of the low FODMAP diet in irritable bowel syndrome: is it ready for prime time as a first‐line therapy? J. Gastroenterol. Hepatol. 2017; 32: 32–35. 28244668 10.1111/jgh.13693

[7] Black CJ , Staudacher HM , Ford AC . Efficacy of a low FODMAP diet in irritable bowel syndrome: systematic review and network meta‐analysis. Gut. 2022; 71: 1117–1126. 34376515 10.1136/gutjnl-2021-325214

[8] Gibson PR , Halmos EP , Muir JG . Review article: FODMAPS, prebiotics and gut health‐the FODMAP hypothesis revisited. Aliment. Pharmacol. Ther. 2020; 52: 233–246. 32562590 10.1111/apt.15818

[9] Wu J , Masuy I , Biesiekierski JR *et al*. Gut‐brain axis dysfunction underlies FODMAP‐induced symptom generation in irritable bowel syndrome. Aliment. Pharmacol. Ther. 2022; 55: 670–682. 35166384 10.1111/apt.16812

[10] Tuck CJ , Abu Omar A , De Palma G *et al*. Changes in signalling from faecal neuroactive metabolites following dietary modulation of IBS pain. Gut. 2022; 72: 1678–1691. 10.1136/gutjnl-2022-327260 36591617

[11] Ajamian M , Rosella G , Newnham ED , Biesiekierski JR , Muir JG , Gibson PR . Effect of gluten ingestion and FODMAP restriction on intestinal epithelial integrity in patients with irritable bowel syndrome and self‐reported non‐coeliac gluten sensitivity. Mol. Nutr. Food Res. 2021; 65: e1901275. 32902928 10.1002/mnfr.201901275

[12] O'Brien L , Kasti A , Halmos EP , Tuck C , Varney J . Evolution, adaptation, and new applications of the FODMAP diet. JGH Open. 2024; 8: e13066. 38770353 10.1002/jgh3.13066 PMC11103764

[13] Singh P , Grabauskas G , Zhou SY , Gao J , Zhang Y , Owyang C . High FODMAP diet causes barrier loss via lipopolysaccharide‐mediated mast cell activation. JCI Insight. 2021; 6: 20211122. 10.1172/jci.insight.146529 PMC8663790 34618688

[14] Zhou SY , Gillilland M 3rd , Wu X *et al*. FODMAP diet modulates visceral nociception by lipopolysaccharide‐mediated intestinal inflammation and barrier dysfunction. J. Clin. Invest. 2018; 128: 267–280. 29202473 10.1172/JCI92390 PMC5749529

[15] Aguilera‐Lizarraga J , Florens MV , Viola MF *et al*. Local immune response to food antigens drives meal‐induced abdominal pain. Nature. 2021; 590: 151–156. 33442055 10.1038/s41586-020-03118-2 PMC7610810

[16] Quigley EMM . Can diet change the natural history of gastrointestinal diseases? JGH Open. 2024; 8: e13063. 38770354 10.1002/jgh3.13063 PMC11103768

[17] So D , Gibson PR , Muir JG , Yao CK . Dietary fibres and IBS: translating functional characteristics to clinical value in the era of personalised medicine. Gut. 2021; 70: 2383–2394. 34417199 10.1136/gutjnl-2021-324891

[18] McKeown NM , Fahey GC Jr , Slavin J *et al*. Fibre intake for optimal health: how can healthcare professionals support people to reach dietary recommendations? BMJ. 2022; 378: e054370. 35858693 10.1136/bmj-2020-054370 PMC9298262

[19] Ford AC , Moayyedi P , Chey WD *et al*. American College of Gastroenterology Monograph on management of irritable bowel syndrome. Am. J. Gastroenterol. 2018; 113: 1–18. 10.1038/s41395-018-0084-x 29950604

[20] McKee DP , Quigley EM . Intestinal motility in irritable bowel syndrome: is IBS a motility disorder? Part 1. Definition of IBS and colonic motility. Dig. Dis. Sci. 1993; 38: 1761–1772. 8404395 10.1007/BF01296097

[21] Gill SK , Rossi M , Bajka B , Whelan K . Dietary fibre in gastrointestinal health and disease. Nat. Rev. Gastroenterol. Hepatol. 2021; 18: 101–116. 33208922 10.1038/s41575-020-00375-4

[22] Lacy BE , Pimentel M , Brenner DM *et al*. ACG Clinical Guideline: Management of Irritable bowel Syndrome. Am. J. Gastroenterol. 2021; 116: 17–44. 33315591 10.14309/ajg.0000000000001036

[23] Moayyedi P , Andrews CN , MacQueen G *et al*. Canadian association of gastroenterology clinical practice guideline for the management of irritable bowel syndrome (IBS). J. Can. Assoc. Gastroenterol. 2019; 2: 6–29. 31294724 10.1093/jcag/gwy071 PMC6507291

[24] Vasant DH , Paine PA , Black CJ *et al*. British Society of Gastroenterology guidelines on the management of irritable bowel syndrome. Gut. 2021; 70: 1214–1240. 33903147 10.1136/gutjnl-2021-324598

[25] Gunn D , Abbas Z , Harris HC *et al*. Psyllium reduces inulin‐induced colonic gas production in IBS: MRI and in vitro fermentation studies. Gut. 2022; 71: 919–927. 34353864 10.1136/gutjnl-2021-324784 PMC8995815

[26] Duncanson K , Burns G , Pryor J , Keely S , Talley NJ . Mechanisms of food‐induced symptom induction and dietary management in functional dyspepsia. Nutrients. 2021; 13: 20210328. 10.3390/nu13041109 PMC8066021 33800668

[27] Goyal O , Nohria S , Batta S , Dhaliwal A , Goyal P , Sood A . Low fermentable oligosaccharides, disaccharides, monosaccharides, and polyols diet versus traditional dietary advice for functional dyspepsia: a randomized controlled trial. J. Gastroenterol. Hepatol. 2022; 37: 301–309. 34555865 10.1111/jgh.15694

[28] So D , Loughman A , Staudacher HM . Effects of a low FODMAP diet on the colonic microbiome in irritable bowel syndrome: a systematic review with meta‐analysis. Am. J. Clin. Nutr. 2022; 116: 943–952. 35728042 10.1093/ajcn/nqac176 PMC9535515

[29] Staudacher HM , Scholz M , Lomer MC *et al*. Gut microbiota associations with diet in irritable bowel syndrome and the effect of low FODMAP diet and probiotics. Clin. Nutr. 2021; 40: 1861–1870. 33183883 10.1016/j.clnu.2020.10.013

[30] Kamal‐Eldin A , Lærke HN , Knudsen KE *et al*. Physical, microscopic and chemical characterisation of industrial rye and wheat brans from the Nordic countries. Food Nutr. Res. 2009; 53: 20090422. 10.3402/fnr.v53i0.1912 PMC2675562 19412350

[31] So D , Yao CK , Gill PA , Pillai N , Gibson PR , Muir JG . Screening dietary fibres for fermentation characteristics and metabolic profiles using a rapid in vitro approach: implications for irritable bowel syndrome. Br. J. Nutr. 2021; 126: 208–218. 33028442 10.1017/S0007114520003943

[32] Bijkerk CJ , de Wit NJ , Muris JW *et al*. Soluble or insoluble fibre in irritable bowel syndrome in primary care? Randomised placebo controlled trial. BMJ. 2009; 339: 20090827. 10.1136/bmj.b3154 PMC3272664 19713235

[33] Remes‐Troche JM , Taboada‐Liceaga H , Gill S *et al*. Nopal fiber (*Opuntia ficus‐indica*) improves symptoms in irritable bowel syndrome in the short term: a randomized controlled trial. Neurogastroenterol. Motil. 2021; 33: e13986. 32935904 10.1111/nmo.13986

[34] So D , Yao CK , Ardalan ZS *et al*. Supplementing dietary fibers with a low FODMAP diet in irritable bowel syndrome: a randomized controlled crossover trial. Clin. Gastroenterol. Hepatol. 2022; 20: 2112–2120.e2117. 34929392 10.1016/j.cgh.2021.12.016

[35] van der Schoot A , Drysdale C , Whelan K , Dimidi E . The effect of fiber supplementation on chronic constipation in adults: an updated systematic review and meta‐analysis of randomized controlled trials. Am. J. Clin. Nutr. 2022; 116: 953–969. 35816465 10.1093/ajcn/nqac184 PMC9535527

[36] Wilson B , Rossi M , Dimidi E , Whelan K . Prebiotics in irritable bowel syndrome and other functional bowel disorders in adults: a systematic review and meta‐analysis of randomized controlled trials. Am. J. Clin. Nutr. 2019; 109: 1098–1111. 30949662 10.1093/ajcn/nqy376

[37] Wilson B , Rossi M , Kanno T *et al*. β‐Galactooligosaccharide in conjunction with low FODMAP diet improves irritable bowel syndrome symptoms but reduces fecal bifidobacteria. Am. J. Gastroenterol. 2020; 115: 906–915. 32433273 10.14309/ajg.0000000000000641

[38] So D , Yao CK , Gill PA *et al*. Detection of changes in regional colonic fermentation in response to supplementing a low FODMAP diet with dietary fibres by hydrogen concentrations, but not by luminal pH. Aliment. Pharmacol. Ther. 2023; 58: 417–428. 37386938 10.1111/apt.17629 PMC10946934

[39] Böhn L , Störsrud S , Törnblom H , Bengtsson U , Simrén M . Self‐reported food‐related gastrointestinal symptoms in IBS are common and associated with more severe symptoms and reduced quality of life. Am. J. Gastroenterol. 2013; 108: 634–641. 23644955 10.1038/ajg.2013.105

[40] Cooke ZM , Resciniti SM , Wright BJ *et al*. Association between dietary factors, symptoms, and psychological factors in adults with dyspepsia: A cross‐sectional study. Neurogastroenterol. Motil. 2023; 35: e14684. 37771208 10.1111/nmo.14684

[41] Sultan N , Varney JE , Halmos EP *et al*. How to implement the 3‐phase FODMAP diet into gastroenterological practice. J. Neurogastroenterol. Motil. 2022; 28: 343–356. 35799231 10.5056/jnm22035 PMC9274476

[42] Felicilda‐Reynaldo RF , Kenneally M . Digestive enzyme replacement therapy: pancreatic enzymes and lactase. Medsurg Nurs. 2016; 25: 182–185. 27522847

[43] Lin MY , Dipalma JA , Martini MC , Gross CJ , Harlander SK , Savaiano DA . Comparative effects of exogenous lactase (beta‐galactosidase) preparations on in vivo lactose digestion. Dig. Dis. Sci. 1993; 38: 2022–2027. 8223076 10.1007/BF01297079

[44] Montalto M , Nucera G , Santoro L *et al*. Effect of exogenous beta‐galactosidase in patients with lactose malabsorption and intolerance: a crossover double‐blind placebo‐controlled study. Eur. J. Clin. Nutr. 2005; 59: 489–493. 15674309 10.1038/sj.ejcn.1602098

[45] Rosado JL , Solomons NW , Lisker R *et al*. Enzyme replacement therapy for primary adult lactase deficiency. Effective reduction of lactose malabsorption and milk intolerance by direct addition of beta‐galactosidase to milk at mealtime. Gastroenterology. 1984; 87: 1072–1082. 6434367

[46] Portincasa P , Di Ciaula A , Vacca M *et al*. Beneficial effects of oral tilactase on patients with hypolactasia. Eur. J. Clin. Invest. 2008; 38: 835–844. 19021701 10.1111/j.1365-2362.2008.02035.x

[47] Corgneau M , Scher J , Ritie‐Pertusa L *et al*. Recent advances on lactose intolerance: tolerance thresholds and currently available answers. Crit. Rev. Food Sci. Nutr. 2017; 57: 3344–3356. 26713460 10.1080/10408398.2015.1123671

[48] Bhosale SH , Rao MB , Deshpande VV . Molecular and industrial aspects of glucose isomerase. Microbiol. Rev. 1996; 60: 280–300. 8801434 10.1128/mr.60.2.280-300.1996 PMC239444

[49] Komericki P , Akkilic‐Materna M , Strimitzer T , Weyermair K , Hammer HF , Aberer W . Oral xylose isomerase decreases breath hydrogen excretion and improves gastrointestinal symptoms in fructose malabsorption ‐ a double‐blind, placebo‐controlled study. Aliment. Pharmacol. Ther. 2012; 36: 980–987. 23002720 10.1111/apt.12057

[50] Tuck CJ , Ross LA , Gibson PR , Barrett JS , Muir JG . Adding glucose to food and solutions to enhance fructose absorption is not effective in preventing fructose‐induced functional gastrointestinal symptoms: randomised controlled trials in patients with fructose malabsorption. J. Hum. Nutr. Diet. 2017; 30: 73–82. 27600184 10.1111/jhn.12409

[51] Tuck CJ , Taylor KM , Gibson PR , Barrett JS , Muir JG . Increasing symptoms in irritable bowel symptoms with ingestion of galacto‐oligosaccharides are mitigated by α‐galactosidase treatment. Am. J. Gastroenterol. 2018; 113: 124–134. 28809383 10.1038/ajg.2017.245

[52] Di Nardo G , Oliva S , Ferrari F *et al*. Efficacy and tolerability of α‐galactosidase in treating gas‐related symptoms in children: a randomized, double‐blind, placebo controlled trial. BMC Gastroenterol. 2013; 13: 20130924. 10.1186/1471-230X-13-142 PMC3849317 24063420

[53] Hillilä M , Färkkilä MA , Sipponen T , Rajala J , Koskenpato J . Does oral α‐galactosidase relieve irritable bowel symptoms? Scand. J. Gastroenterol. 2016; 51: 16–21. 26133538 10.3109/00365521.2015.1063156

[54] Böhn L , Törnblom H , Van Oudenhove L *et al*. A randomized double‐blind placebo‐controlled crossover pilot study: Acute effects of the enzyme α‐galactosidase on gastrointestinal symptoms in irritable bowel syndrome patients. Neurogastroenterol. Motil. 2021; 33: e14094. 33619835 10.1111/nmo.14094

[55] Tuck CJ , Barrett JS , Yao CK . The key to success: targeting enzymes to their dietary counterpart. Neurogastroenterol. Motil. 2021; 33: e14204. 34152051 10.1111/nmo.14204

[56] Guice JL , Hollins MD , Farmar JG , Tinker KM , Garvey SM . Microbial inulinase promotes fructan hydrolysis under simulated gastric conditions. Front. Nutr. 2023; 10: 1129329. 37305092 10.3389/fnut.2023.1129329 PMC10251236

[57] Ochoa KC , Samant S , Liu A *et al*. In vitro efficacy of targeted fermentable oligosaccharides,disaccharides, monosaccharides, and polyols enzymatic digestion in a high‐fidelity simulated gastrointestinal environment. Gastro. Hep. Adv. 2023; 2: 283–290. 10.1016/j.gastha.2022.10.011 PMC11308120 39132653

[58] Pham VT , Steinert RE , Duysburgh C , Ghyselinck J , Marzorati M , Dekker PJT . In vitro effect of enzymes and human milk oligosaccharides on FODMAP digestion and fecal microbiota composition. Nutrients. 2023; 15: 20230328. 10.3390/nu15071637 PMC10097142 37049481

[59] Zheng T , Eswaran S , Photenhauer AL , Merchant JL , Chey WD , D'Amato M . Reduced efficacy of low FODMAPs diet in patients with IBS‐D carrying sucrase‐isomaltase (SI) hypomorphic variants. Gut. 2020; 69: 397–398. 30658996 10.1136/gutjnl-2018-318036 PMC6984052

[60] Treem WR , McAdams L , Stanford L , Kastoff G , Justinich C , Hyams J . Sacrosidase therapy for congenital sucrase‐isomaltase deficiency. J. Pediatr. Gastroenterol. Nutr. 1999; 28: 137–142. 9932843 10.1097/00005176-199902000-00008

[61] Foley A , Halmos EP , Husein DM *et al*. Adult sucrase‐isomaltase deficiency masquerading as IBS. Gut. 2022; 71: 1237–1238. 34615726 10.1136/gutjnl-2021-326153 PMC9120380

[62] Krishnareddy S , Stier K , Recanati M , Lebwohl B , Green PHR . Commercially available glutenases: a potential hazard in coeliac disease. Therap. Adv. Gastroenterol. 2017; 10: 473–481. 10.1177/1756283X17690991 PMC5424869 28567117

[63] Salden BN , Monserrat V , Troost FJ *et al*. Randomised clinical study: Aspergillus Niger‐derived enzyme digests gluten in the stomach of healthy volunteers. Aliment. Pharmacol. Ther. 2015; 42: 273–285. 26040627 10.1111/apt.13266 PMC5032996

[64] Lähdeaho ML , Kaukinen K , Laurila K *et al*. Glutenase ALV003 attenuates gluten‐induced mucosal injury in patients with celiac disease. Gastroenterology. 2014; 146: 1649–1658. 24583059 10.1053/j.gastro.2014.02.031

[65] Ferrone M , Raimondo M , Scolapio JS . Pancreatic enzyme pharmacotherapy. Pharmacotherapy. 2007; 27: 910–920. 17542772 10.1592/phco.27.6.910

[66] Sikkens EC , Cahen DL , Kuipers EJ *et al*. Pancreatic enzyme replacement therapy in chronic pancreatitis. Best Pract. Res. Clin. Gastroenterol. 2010; 24: 337–347. 20510833 10.1016/j.bpg.2010.03.006

[67] Domínguez‐Muñoz JE . Pancreatic enzyme replacement therapy for pancreatic exocrine insufficiency: when is it indicated, what is the goal and how to do it? Adv. Med. Sci. 2011; 56: 1–5. 21450558 10.2478/v10039-011-0005-3

[68] Domínguez‐Muñoz JE , Iglesias‐García J , Iglesias‐Rey M , Figueiras A , Vilariño‐Insua M . Effect of the administration schedule on the therapeutic efficacy of oral pancreatic enzyme supplements in patients with exocrine pancreatic insufficiency: a randomized, three‐way crossover study. Aliment. Pharmacol. Ther. 2005; 21: 993–1000. 15813835 10.1111/j.1365-2036.2005.02390.x

[69] Trang T , Chan J , Graham DY . Pancreatic enzyme replacement therapy for pancreatic exocrine insufficiency in the 21(st) century. World J. Gastroenterol. 2014; 20: 11467–11485. 25206255 10.3748/wjg.v20.i33.11467 PMC4155341

[70] Menezes LAA , Molognoni L , de Sá Ploêncio LA , Costa FBM , Daguer H , Dea Lindner JD . Use of sourdough fermentation to reducing FODMAPs in breads. Eur. Food Res. Technol. 2019; 245: 1183–1195.

[71] Sharp E , D'Cunha NM , Ranadheera CS , Vasiljevic T , Panagiotakos DB , Naumovski N . Effects of lactose‐free and low‐lactose dairy on symptoms of gastrointestinal health: a systematic review. Int. Dairy J. 2021; 114: 104936.

[72] Li Q , Loponen J , Gänzle MG . Characterization of the extracellular fructanase FruA in *Lactobacillus crispatus* and its contribution to fructan hydrolysis in breadmaking. J. Agric. Food Chem. 2020; 68: 8637–8647. 32687341 10.1021/acs.jafc.0c02313

[73] Nyyssölä A , Nisov A , Lille M *et al*. Enzymatic reduction of galactooligosaccharide content of faba bean and yellow pea ingredients and food products. Fut. Foods. 2021; 4: 100047.

[74] Ispiryan L , Zannini E , Arendt EK . FODMAP modulation as a dietary therapy for IBS: scientific and market perspective. Compr. Rev. Food Sci. Food Saf. 2022; 21: 1491–1516. 35122383 10.1111/1541-4337.12903

